# Development and evaluation of an undergraduate curriculum on non-communicable disease research in Guam: The Pacific Islands Cohort of College Students (PICCS)

**DOI:** 10.1186/s12889-021-12078-9

**Published:** 2021-11-03

**Authors:** Yvette C. Paulino, Anthony Ada, John Dizon, Elisha-Rose J. Benavente, Katherine Mary De Luna Campbell, Breinard Cristobal, Alexandria Daughtry, Lorenz Michael O. Estabillo, Victoria Diana Cruz Flisco, Grazyna Badowski, Margaret Hattori-Uchima

**Affiliations:** 1 University of Guam, School of Health, University Drive, Mangilao, GU 96923 USA; 2 University of Guam, College of Natural & Applied Sciences, Biology Program, University Drive, Mangilao, GU 96923 USA; 3 University of Guam, College of Natural & Applied Sciences, Mathematics Program, University Drive, Mangilao, GU 96923 USA

**Keywords:** Curriculum development, Data for decision making, Epidemiology, Health disparities, Minority health, Native Hawaiian or other Pacific Islander, Non-communicable diseases, Public health, Undergraduate research, US-Affiliated Pacific Islands

## Abstract

**Background:**

The non-communicable disease (NCD) epidemic among Pacific Islanders prompted the declaration of a regional state of NCD emergency throughout the United States-Affiliated Pacific Islands (USAPIs) in 2010. Subsequently, the University of Guam Health Science Program launched a pilot study on NCD research in its undergraduate curriculum modeled after the Pacific Data for Decision Making (DDM) framework – a field epidemiology training program employed in the USAPIs. The primary objective of the research is to conduct annual assessments of student health indicators with plans for longitudinal follow-up. Here, development and evaluation of the undergraduate research curriculum are described.

**Methods:**

The Pacific DDM framework covering knowledge and skills in resourcing, types of data and indicators, data sources, data management, information products, and data dissemination and use were incorporated in undergraduate core courses of the Health Science Program. During the data collection pilot years, 2013 and 2014, a survey containing questions predominantly on NCD risk factors was launched at the university. The survey was administered by upperclassmen in the Health Science Program and evolved into the Pacific Islands Cohort of College Students (PICCS) research study. The initial years were spent developing the infrastructure. Program outputs were tracked annually to measure program success.

**Results:**

Students in the Health Science Program obtained research knowledge and skills through various courses while enrolled in the program. The PICCS data collection continued annually as a cross-sectional survey from 2015 to current. Numerous successes have resulted including student abstracts and publications, acceptances to summer programs and fellowships, a sustained annual health fair for college students, a grant award, and other program-related impacts.

**Conclusion:**

The PICCS framework provided the organizational structure and documented tools, protocols, roles, and responsibilities to enhance consistency and reproducibility. Undergraduate students applied their knowledge and skills to an ongoing study focused on NCD risk factor surveillance of college students. Additionally, multiple research successes have been achieved through the PICCS curriculum. Plans are underway to begin the longitudinal design of the PICCS research study and sustain it through the curriculum, with room for adaptation as courses are updated over time.

## Background

Non-communicable diseases (NCDs), including cardiovascular diseases, cancer, respiratory diseases, and diabetes, remain among the leading causes of death globally. Combined NCDs account for 71% (41 of 57 million) of all deaths [1], with a projected increase to 52 million by 2030. In the Pacific region, 40% of the 9.7 million residents endure NCDs while 75% of deaths are NCD-related [2]. To mitigate NCDs in the Pacific, stakeholders developed and circulated the NCD Roadmap suggesting a multisectoral approach to implement four key strategies: control tobacco use through increased taxes, reduce consumption of foods and drinks linked to NCDs through policies, improve efficiency and impact, and improve evidence base for decision making [3]. Monitoring and surveillance of NCD-related health indicators have been prioritized through the Pacific Monitoring Alliance for NCD Action framework in at least 22 Pacific Island countries and territories, including those affiliated with the United States (US) [4].

The US-Affiliated Pacific Islands (USAPIs) are home to an estimated half-million people spread across Oceania including the islands of American Samoa, Guam, Commonwealth of Northern Marianas, Republic of the Marshalls, Republic of Palau, and the Federated States of Micronesia (Pohnpei, Chuuk, Kosrae and Yap). The median age of USAPI residents ranges from 24 years old in the Republic of the Marshall Islands to 34 years old in the Republic of Palau, while life expectancy ranges from 74 years old in the Federated States of Micronesia to 77 years old in Guam [5]. The USAPIs experience some of the highest poverty in the US, as high as 41% in the Federated States of Micronesia [5]. Additionally, chronic diseases and risk factors are prevalent in the USAPIs [6–15], with early obesity [16], food insecurity [17], and use of tobacco [18] and areca nut [19–21] among the major drivers.

### Response to NCDs in the USAPIs

Ministers and directors of health from the USAPIs form the Pacific Islands Health Officers’ Association (PIHOA). In 2010, PIHOA declared a Regional State of Health Emergency due to the NCD epidemic in the USAPI through Board Resolution #48–01 [22]. Prior to the resolution, the Pacific Chronic Disease Council (PCDC) developed a Pacific NCD Collaborative Initiative assessing the USAPIs’ NCD-related services and expanding the population outreach [23]. Subsequently, Board Resolution #48–01 reinforced the development of an NCD strategic plan among the USAPIs. PIHOA guided the islands on development and implementation of their strategic plan through participation in an epidemiology training program for Pacific health workforce [24] adopted from the Pacific Data for Decision Making (DDM) Program [25]. The primary objective of the Pacific Islands Cohort of College Students (PICCS) research is to conduct annual assessments of NCD health risk factors of students enrolled at the University of Guam (UOG), with plans for a long-term longitudinal follow-up. In this paper, the development and evaluation of the undergraduate research curriculum are described.

## Methods

### Pacific Islands Cohort of College Students (PICCS) curriculum

The Pacific DDM framework, which aligns with the Health Metrics Framework, incorporates six major components of health data utilization to inform decision-making. The components include resourcing, types of data and indicators, data sources, data management (data collection, data entry and storage, data cleaning, data analysis and interpretation, organizing and presenting data), information products, and data dissemination and use [24]. During 2013 and 2014, the UOG Health Science Program adopted components of the Pacific DDM framework as it began exploring a sustainable approach to undergraduate research. A pilot survey of health questions predominantly on NCD risk factors was launched. Individuals eligible to participate in the survey were students currently attending the university and at least 18 years of age. The survey was administered by upperclassmen and evolved into the student-led PICCS research study.

The Pacific DDM concepts and the PICCS activities were phased into selected Health Science Program core courses described in Table 1. The insertion of population health research-related themes such as health assessment, study design, data collection and analysis, dissemination, translation, and application were scaffolded from sophomore level courses to senior level courses. Specifically, students were introduced to anthropometry (HS200), NCD risk factor assessment (HS216), study design and survey development (HS405), PICCS data collection (HS416), analysis (MA387), dissemination (HS451), translation (HS491), and application (HS498). These curricular concepts corresponded to components of the Pacific DDM framework.

**Table 1:** Selected core courses supporting the Pacific Islands Cohort of College Students (PICCS) curriculum of the Bachelor of Science Health Science Program, University of Guam

Course	Content	Corresponding Pacific DDM Component
HS200 Health and Wellness	Exposure to PICCS anthropometry	Data management (collection tools)
HS216 Introduction to Public Health	Exposure to PICCS assessment of NCD risk factors	Resourcing, types of data and indicators
HS405 Epidemiology	Exposure to PICCS study design and methodology	Types of data and indicators, data sources
HS416 Research in Health Sciences	Collection of PICCS data	Data management
MA387 Statistics for Sciences	Analysis of PICCS data	Data management
HS451 Research and Report Writing	Dissemination of PICCS findings	Information products, data dissemination and use
HS491 Current Topics in Health Sciences	Translation of PICCS and other health topics	Data management, information products, data dissemination and use
HS498 Internship in Health Sciences	Application of PICCS and other program knowledge and skills	Resourcing, types of data and indicators, data sources, data management, information products, data dissemination and use

### Research study

The PICCS research study has been incorporated in the Health Science Program. Data collection was piloted in 2013 to 2014 in the HS416 course and conducted annually thereafter as a cross-sectional survey on the health status of college students. Initial ethics approval was obtained in 2013 (CHRS#13–96) from the Committee on Human Research Subjects at the University of Guam and remains current (CHRS#20–121). Recruitment for participation in the PICCS research study was held every Fall semester in HS416 from September to December. Individuals eligible to participate in the survey were students at least 18 years of age and currently attending the university. Over the years, participants were recruited through announcements via word-of-mouth, flyers posted throughout campus, social media, and the university’s student email list. Except for Fall 2020 due to the coronavirus pandemic, face-to-face interviews were conducted on campus in a designated research room. All the study materials and equipment were kept secured in locked cabinets or by locking the room accessible only to two research teams.

**Fig. 1:**
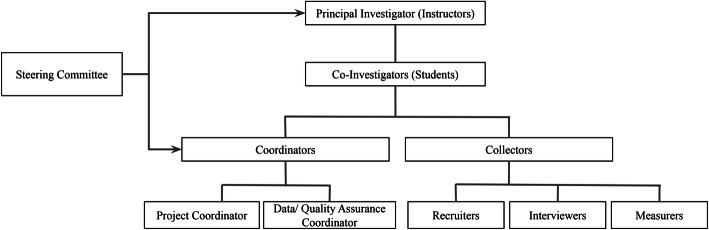
Organizational framework of the data collection team of the Pacific Islands Cohort of College Students (PICCS) curriculum of the Bachelor of Science Health Science Program, University of Guam

Years 2015 to 2017 were spent developing the processes and establishing the infrastructure according to Fig. 1. Instructors remained principal investigators (PI) throughout the project. During junior and senior year, student researchers were upgraded to co-investigators and assumed one of the following roles: 1) Coordinator - *Project Coordinator* (assisted PI with trainings, scheduling, operations, logistics) or *Data/Quality Assurance Coordinator* (assisted PI with standardization, equipment calibration, survey completion); or 2) Collector – *Recruiter* (sought participants), *Interviewer* (administered questionnaires), or *Measurer* (collected body measurements). All student co-investigators practiced role-playing the informed consent process and administering the questionnaires several times before recruitment commenced each year. Measurers were trained and standardized to collect height and weight measurements using similar protocols from the Children’s Healthy Living (CHL) Program [26]. Measurers who did not pass the standardization were encouraged to select from other co-investigator roles.

### Data collection

Initial health indicators were selected by research students in 2013 and 2014 based on the Healthy People 2020 topics taught in the HS200 course, perception of relevance, and availability of tools. Subsequently, indicators were added or modified as seen in Table 2 by request or to reflect changes to other local surveys such as the Guam Behavioral Risk Factor Surveillance System (BRFSS), the CHL Program, and the University of Guam/University of Hawai'i Cancer Center Partnership to Advance Cancer Health Equity (PACHE). Survey administration was conducted in pairs (interviewer and measurer) after the pilot years. Data collected consisted of physical measurements and self-reported information. The physical data included: body fat measured in percentage with the Omron Fat Loss Monitor, Model HBF-306C/Black (Omron Healthcare Inc., Lake Forest, Illinois), height measured in centimeters (cm) with a portable stadiometer (Model PE-AIM-101, Perspective Enterprises, Portage, Michigan), weight measured in kilograms with a portable weight scale (Seca 876, Hamburg, Germany), waist and neck circumferences measured in cm with plastic tape (Seca Model 201, Chino, California), blood pressure measured in millimeters of mercury (mmHg) manually (with a blood pressure cuff, sphygmomanometer, and stethoscope) and digitally (with automated blood pressure machine), and acanthosis nigricans [27]. The measurer performed the anthropometry and reported the numbers for the interviewer to record. Measurements continued until two consecutive measurements were within ±0.2 units, except for blood pressure which was measured at least twice regardless of reading.

**Table 2:** Health indicators measured among students enrolled in the Pacific Islands Cohort of College Students (PICCS) from 2015 to 2019, University of Guam

Health Indicators	Years Collected
Anthropometry, acanthosis nigricans, and blood pressure	2015–2016 (body fat)
	2015–2019 (height, weight)
	2017–2018 (waist circumference)
	2017–2019 (acanthosis nigricans)
	2017, 2019 (blood pressure)
	2019 (neck circumference)
Background information	2015–2019 (demographics)
	2017–2019 (culture)
Food, nutrition, and physical activity	2015–2019 (fruit, vegetable, fast food, physical activity, water)
	2017–2019 (food security, spicy food)
Health care	2015–2019 (health care access, personal medical history)
Mental health	2015–2019 (sleep, stress)
	2017–2019 (anxiety, depression)
Occupational and environment	2017–2019
Oral health	2015–2016, 2019 (areca nut, tobacco, dental visit)
	2015–2019 (alcohol)
	2016–2019 (e-cigarettes, marijuana)
Sexually transmitted infections	2015–2019 (awareness)

Self-reported data included background information [28] (demographics and culture), food security and spicy food intake [28], areca nut use [29], sexually transmitted infections (STIs) awareness [30], physical activity [20, 31], personal medical history, sleep [32], stress [33], anxiety and depression [34], occupational and environmental exposure [35], and questions adopted from the BRFSS including alcohol, dental visits, e-cigarettes, fast food consumption, fruit and vegetable consumption, health care access, medical marijuana, and tobacco use [36].

### Evaluation

Outputs of the Health Science Program were tracked annually to measure the success of the PICCS curriculum. Tracked outputs included the number of PICCS-related abstracts and publications, acceptances to summer programs and fellowships, annual health fair activities, contributions to external research funding, and overall program impact (number of Health Science student internships and graduates with PICCS research experience). Additionally, the student researchers submitted comments on their experiences during HS451, the final research course in the program.

## Results

The Health Science Program successes related to the PICCS from the years 2015 to 2020 are listed in Table 3. In addition to the successes tracked, the student researchers provided positive comments and suggested areas for improvement. The positive comments included an appreciation for enhanced research skills and the opportunity to engage stakeholders:

*“My research skills have actually improved … searching for and citing peer reviewed articles.”*

*“I used software to create visual data in a way that would be legible to Non-Health Science majors to ensure they would be able to interpret …*”.

*“Another good experience was that we had a feedback loop with our instructor in which we submitted our research and feedback would be given to us promptly … This process happened multiple times … in the research and writing process.”*

*“The HART Fair gave us a chance to be part of an activity that benefits a population. These are skills many of us will use when performing outreach in the community post-graduation.”*

**Table 3:** Health Science Program successes related to the Pacific Islands Cohort of College Students (PICCS) curriculum, 2015 to 2020

Year	Number of Undergraduates	Successes
2015	232	28 student internships with government and non-government organizations 28 graduates with Bachelor of Science in Health Science Two conference abstract presentations One acceptance to public health scholars summer program Leadership role on Guam Non-Communicable Disease Consortium
2016	218	24 student internships with government and non-government organizations 24 graduates with Bachelor of Science in Health Science Three conference abstract presentations One acceptance to public health scholars summer program One acceptance to child health assessment summer fellowship One 5-year research grant award 1st Annual Health Awareness to Reach Tritons Fair Leadership role on Guam Non-Communicable Disease Consortium
2017	237	27 student internships with government and non-government organizations 27 graduates with Bachelor of Science in Health Science One conference abstract presentation One conference panel presentation One acceptance to child health assessment summer fellowship 2nd Annual Health Awareness to Reach Tritons Fair Leadership role on Guam Non-Communicable Disease Consortium Addition of 1-credit computer laboratory to MA387 statistical course Village health profiles for Mayors’ Council of Guam
2018	263	26 student internships with government and non-government organizations 26 graduates with Bachelor of Science in Health Science Two conference abstract presentations Two conference panel presentations One acceptance to child health assessment summer fellowship 3rd Annual Health Awareness to Reach Tritons Fair Leadership role on Guam Non-Communicable Disease Consortium
2019	238	21 student internships with government and non-government organizations 21 graduates with Bachelor of Science in Health Science Six conference abstract presentations 4th Annual Health Awareness to Reach Tritons Fair Leadership role on Guam Non-Communicable Disease Consortium
2020	215	24 student internships with government and non-government organizations 24 graduates with Bachelor of Science in Health Science One manuscript publication One acceptance to public health scholars summer program 5th Annual Health Awareness to Reach Tritons Fair Leadership role on Guam Non-Communicable Disease Consortium

The suggested areas for improvement were directed towards enhancing dissemination.

*“Increase the number of classes involved in hosting the HART Fair … nursing class, psychology class …”.*

*“Invite other student organizations and challenge them to focus on health, mental health, wealth, civics, and social justice (all of which pertain to health).”*

*“Provide clear criteria and evaluation of group contributions.”*

*“Transitioning from face-to-face to online event during the pandemic was stressful at first … should incorporate more committee meetings...consider expanding presence on social media platforms.”*

The first category of program successes listed in Table 3 is student internships with various government and non-government organizations. The internships corresponded with the number of graduates as internships were performed during the semester of graduation. Over 200 undergraduates were enrolled in the program each year, with a range of 9 to 12% graduating each year. Other successes included a total of 17 student and faculty abstract presentations at conferences, one research grant award from the National Institute on Minority Health and Health Disparities (NIMHD) under the National Institutes of Health (NIH) (Grant Number 5U24MD011201), development of a 1-credit computer laboratory course added to the MA387 statistics course to enhance students’ statistical skills in research, creation of the village health profiles for 19 villages and shared with the mayors of the villages, and one student-led manuscript publication [37]. Three students were selected to participate in the Centers for Disease Control and Prevention Undergraduate Public Health Scholars (CUPS) Program [38], but unlike the CUPS, the PICCS curriculum engages students over multiple undergraduate years. Three other students were selected to participate in the Child Health Assessment in the Pacific Undergraduate Summer Fellowship Program [39]. Over the years, the program has maintained a leadership role on the Data and Surveillance Team of the Guam Non-Communicable Disease Consortium and continued to host the annual Health Awareness to Reach Tritons (HART) Fair, which is an event initiated by students from the 2016 class to share the research results with their fellow Tritons (UOG’s mascot). Although not an intended outcome, the HART Fair evolved into a space for linking students to disease prevention activities (e.g., nutrition education booths, physical activity competition, meditation demonstrations) and free services (e.g., access to tobacco prevention and cessation coordinators, access to tests for sexually transmitted infections). The impact of this linkage on NCD prevention among the college students may be further explored.

## Discussion

### PICCS as a sustainable undergraduate training for research

Students majoring in the Health Science Program at the University of Guam beginning 2013 received training on the knowledge and skills pertinent to research ethics, planning, implementation, analysis, dissemination, and translation of the PICCS study through core courses described in Table 1 and reinforced in other courses required of the program. The incorporation of PICCS activities into the program courses encourages sustained undergraduate research experiences, with room for adaptation as courses are updated over time. Students’ suggestions for improvement would be considered with course updates. For example, the PICCS learning outcomes could be aligned with those of courses from other interested disciplines across campus. Criteria and evaluation of collaborative group efforts will be articulated.

The engagement of undergraduates in research has been a practice for many years. The US National Science Foundation first launched an undergraduate research program in 1958, and two decades later the Council on Undergraduate Research was formed [40]. There are many programs promoting undergraduate research experiences, especially in the fields of science, technology, engineering, and mathematics [40], and some are now promoting course-based undergraduate research experiences to reach undergraduates in masses [41]. The PICCS curriculum may be considered an expansion of this course-based undergraduate research experiences, with the research experience sustained through the PICCS research study targeting college students. The university presents a unique space for young adults to lead research targeting their peers and translate findings into meaningful outcomes. In 2013, the average age of undergraduates at the University of Guam was 23 years old [42] which is in the age range of when the propensity for health risk taking, such as drinking alcohol and smoking cigarettes, is heightened [43]. Fortunately, young adults are in a period of their life when “mistakes and failures can be reversed” and they are “responsive to education and training and to incentives to create and contribute.” [44] Thus, the PICCS curriculum offers a critical window of opportunity for health risk interventions including education, training, and innovation among young adults in college.

### Future plans

Plans are underway to begin the longitudinal cohort design of the PICCS. Research participants that consented to be recontacted will be invited to participate in the follow-up. The PICCS team will develop additional tools, methods, and processes, including the follow-up schedule, in preparation of the NCD cohort of college students.

## Conclusion

NCD morbidity and mortality are problematic even in the remote areas of the Pacific where challenges are further exacerbated by distance and limited resources. Through the PICCS curriculum, students learn research and apply their knowledge and skills to an ongoing study focused on NCD risk factor surveillance of college students. Incorporation of the PICCS in an undergraduate curriculum provides a unique opportunity for young adults to lead research targeting their peers. The PICCS framework provides the organizational structure and documented tools, protocols, roles, and responsibilities to ensure consistency and reproducibility. Plans are underway to begin the prospective follow-up design of the PICCS research study, which can be sustained through the curriculum with room for adaptation as courses are updated over time.

## Data Availability

Access to the PICCS materials can be requested through the University of Guam, given compliance with ethical review requirements. Contact the corresponding author at paulinoy@triton.uog.edu.

## Abbreviations

PICCS: Pacific Islands Cohort of College Students
NCDs: Non-communicable diseases
DDM: Pacific Data for Decision Making Program
US: United States
USAPIs: United States-Affiliated Pacific Islands
PIHOA: Pacific Health Officers’ Association
PCDC: Pacific Chronic Disease Council
HS200: Health and Wellness
HS216: Introduction to Public Health
HS405: Epidemiology
HS416: Research in Health Sciences
MA387: Statistics for Sciences
HS451: Research and Report Writing
HS491: Current Topics in Health Sciences
HS498: Internship in Health Sciences
UOG: University of Guam
PI: Principal investigator
cm: Centimeters
mmHg: Millimeters of mercury
CHL: Children’s Health Living (CHL) Program
PACHE: Partnership to Advance Cancer Health Equity
STIs: Sexually transmitted infections
BRFSS: Behavioral Risk Factor Surveillance System
NIMHD: National Institute on Minority Health and Health Disparities
NIH: National Institutes of Health
CUPS: Centers for Disease Control and Prevention Undergraduate Public Health Scholars Program
HART: Health Awareness to Reach Tritons Fair
